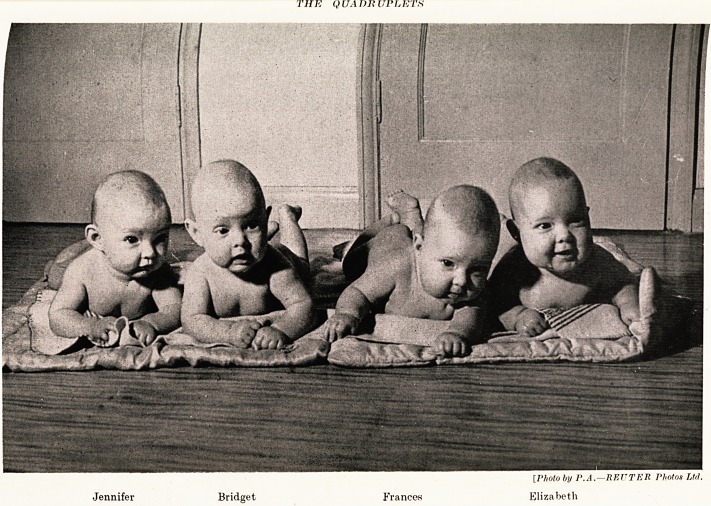# The Quadruplets

**Published:** 1949-01

**Authors:** H. L. Shepherd, Mabel F. Potter, Beryl D. Corner, E. J. Watson-Williams

**Affiliations:** Obstetrician, Bristol Royal Hospital Visiting Obstetrician, Southmead Hospital; Obstetrician, Bristol Royal Hospital Visiting Obstetrician, Southmead Hospital; Physician, Bristol Royal Hospital for Sick Children; Pædiatrician, Southmead Hospital; Sometime obstetric clerk, Southmead Hospital


### THE QUADRUPLETS: I. DELIVERY

BY

H. L. Shepherd, Ch.M., F.R.C.O.G. and

Mabel F. Potter, M.D., F.R.C.O.G.

*Obstetricians, Bristol Royal Hospital*

*Visiting Obstetricians, Southmead Hospital.*

---

Mrs. M. G. was admitted to Southmead Hospital on 10th June, 1948, with well-marked symptoms of toxæmia of pregnancy. She was a tall, well-built woman of twenty-seven. There was one daughter aged two, born after a normal pregnancy and labour.* Mrs. G.'s last menstrual period had been on 10th October, 1947. All through this pregnancy her abdominal enlargement had been unusually great and a multiple pregnancy had been suspected, but a skiagram taken at the end of March had shown only one fœtus.

On admission, her blood pressure, which had been observed to be rising during the previous three weeks, was 170/100. There was marked œdema and albuminuria. The girth of the abdomen was 47½ inches and palpation strongly suggested a triple pregnancy. A skiagram taken on the morning of 11th June quite clearly showed four fœtal heads.

There was a definite increase in the œdema and albuminuria during the next twenty-four hours, accompanied by a further rise in the blood pressure to 180/105, with severe headache and blurring of vision. This rapid increase in symptoms suggested the wisdom of a rapid delivery. A classical Cæsarean section was therefore performed under cyclopropane anæsthesia. Apart from some difficulty in rupturing the fourth sac this presented no unusual problems. The patient made a rapid recovery, the toxæmic symptoms disappearing within three days.

[An illustrated account of this is published in *The Nursing Times 17.7.48. XLIV, 29.*]

* For further family history see "Notes," p. 18.

### THE QUADRUPLETS: II. MANAGEMENT

BY

Beryl D. Corner, M.D., M.R.C.P.

*Physician, Bristol Royal Hospital for Sick Children;*

*Pædiatrician, Southmead Hospital.*

---

On the morning of 12th July, 1948, the Premature-Baby Unit staff at Southmead Hospital was informed that quadruplets were to be delivered by Cæsarean section at 2.0 p.m. Four "Sorrento" type premature-baby cots were prepared, heated to 90° F. and fitted with oxygen tents. The first three babies, Bridget, Frances

PLATE III

THE QUADRUPLETS: X-RAY FILM

The arrows point to outline of fourth head and Elizabeth, cried immediately on delivery, were wrapped in warm sterile towels and placed in their cots. The fourth child, Jennifer, was in a state of blue asphyxia which persisted for four minutes. About one and a half ounces of fluid were aspirated from her respiratory passages by a rubber mucous catheter and laryngoscope, and a stream of oxygen was directed into her pharynx before she gasped and shortly afterwards started regular respiration. All babies were given Synkavit 1 c.cm. intramuscularly and then transferred to the Premature Unit, where the air temperature is maintained at 75° F. and humidity 65 per cent.

During the first twelve hours occasional aspiration of mucus was necessary, but the children cried well and gave rise to little anxiety. The usual premature-baby nursing and feeding technique was employed, which included turning the infants two-hourly in their cots and giving oxygen with 7 per cent. carbon dioxide inhalations at the same time to stimulate respiration. The use of oxygen tents was discontinued on the seventh day. Minimum skin cleaning was carried out with sterile liquid paraffin, and the babies were clothed in flannel gowns and hoods.

Feeding was begun after twelve hours with sterile water, one drachm by bottle two-hourly. Although at first sucking was satisfactory, on the second day there was a tendency to regurgitate, and therefore four-hourly feeding by œsophageal tube with diluted human milk (quarter-strength) was employed. Bottle feeding was resumed three-hourly on the fourth day, giving eight feeds in twenty-four hours. The strength and quantity of human milk was increased daily till on the seventh day the children were taking two ounces per pound of body weight. Owing to the tendency to relaxed stools full-strength milk was not given until the ninth day. By the fourteenth day three ounces per pound of body weight was taken in seven feedings daily, some of the milk being supplied by the mother, the remainder being pooled human milk.

At the end of the third week the decision was made that, in view of the psychological and practical problems involved in singling out one child for breast feeding, human milk should be gradually discontinued. Frailac was therefore introduced slowly, with some added protein as skimmed dried milk for the three bigger children and casein hydrolysate 2 per cent. for Bridget. At six weeks all babies were fully fed on Frailac, three ounces per pound of body weight daily, with protein additions. During the subsequent fortnight a gradual changeover was made to "Special" half-cream "Cow and Gate" dried milk (protein 3·4 per cent., fat 1·8 per cent., lactose 4·9 per cent.), to which was added half a drachm of cane-sugar in each feed, and the number of feeds was reduced to six daily. At eight weeks all babies were receiving approximately sixty calories per pound per day.

During the twelfth week full-cream "Cow and Gate" milk feeds were begun, so that the complete change was effected by fourteen weeks, and, at the same time, the two larger children were reduced to five feeds daily (seven ounces per feed), cane-sugar one drachm and glucose half a drachm being added to each feed.

Owing to the considerable risk of vitamin and iron deficiencies occurring, three supplements were introduced early. Ascorbic acid 10 mgm. daily was started on the seventh day and increased daily up to 50 mgm. by the fourteenth day. High potency vitamin D and calcium (900 units vit. D) was started at the same time and increased up to 3,600 units vitamin D after three weeks. Neoferrum minim i daily was begun at four weeks and increased to minims v at sixteen weeks. The hæmoglobin has been estimated weekly from the seventh day and has remained well up to normal levels.

During the third week of life the children were transferred to a cooler nursery, temperature 65°–70° F., and were placed on the ward balcony, weather permitting. On the ninety-sixth day they were discharged from hospital to two "farm-worker" type council houses specially adapted for the family. The infants were nursed throughout their stay in hospital by the staff of the Premature-Baby Unit, supplemented in the later stages by two nurses who had previously worked in the unit and who went home with them to continue their care.

TABLE I

WEIGHT

| | *Birth* | | 7 Days | | 12 Days | | 48 Days | | 96 Days | | 133 Days | |
|---|---|---|---|---|---|---|---|---|---|---|---|---|
| | lb. | oz. | lb. | oz. | lb. | oz. | lb. | oz. | lb. | oz. | lb. | oz. |
| 1. Bridget .. | 3 | 13 | 3 | 9½ | 3 | 14 | 5 | 10 | 8 | 14½ | 12 | 1½ |
| 2. Frances .. | 4 | 0¼ | 3 | 11½ | 3 | 15 | 6 | 2 | 9 | 8½ | 13 | 1 |
| 3. Elizabeth | 4 | 8 | 4 | 4 | 4 | 10 | 6 | 8 | 9 | 15 | 13 | 5 |
| 4. Jennifer .. | 3 | 14 | 3 | 10 | 3 | 14 | 5 | 13 | 8 | 14 | 12 | 3½ |

TABLE II

HÆMOGLOBIN PERCENTAGES

| | 7 Days | 21 Days | 35 Days | 84 Days | 121 Days |
|---|---|---|---|---|---|
| 1. Bridget .. | 140 | 116 | 100 | 80 | 88 |
| 2. Frances .. | 140 | 104 | 84 | 88 | 92 |
| 3. Elizabeth .. | 152 | 135 | 120 | 86 | 96 |
| 4. Jennifer .. | 136 | 115 | 110 | 92 | 90 |

*Postscript.* February 9th. Bridget has cut her first tooth.

PLATE IV

[Photo by P.A.—REUTER Photos Ltd.

Jennifer Bridget Frances Elizabeth

### THE QUADRUPLETS. III.—NOTES

BY

E. J. WATSON-WILLIAMS, B.A.

*Sometime obstetric clerk, Southmead Hospital.*

---

*The placenta* is large, measuring 36 x 23 cm. It consists of a main part, roughly circular with a diameter of 23 cm., attached to which is a somewhat quadrilateral portion about 15 x 13 cm.: a well-marked groove on both faces separating the two. One cord is attached to the smaller and thinner portion. The area of the larger part remote from the smaller (amounting to about one-third) is marked off by a slight straight ridge arranged chordwise. Two umbilical cords are attached to the central, largest, part at some distance from each other, the fourth springing from the smaller division of the large circular mass. The membranes consist of a separate chorion and amnion for the quadrilateral portion (probably belonging to the infant delivered last); a chorion including two amniotic sacs for the central portion, and a chorion and amnion for the small division of the large part, the chorion of this apparently fused with the middle chorion where these are in contact. It may be inferred that these four children result from fertilization of three ova, the middle pair being "identical" or uniovular twins.

I wish to thank Mr. H. L. Shepherd for permission to use this material.

*Frequency.* The remarkable feature of this case is that quadruplets were carried so near full term. Toxæmia of pregnancy, premature labour, and fœtal mortality are considerably greater in twin than in single pregnancy, very much more with triplets, etc. The frequency of multiple births is shown in the table.

WHITE BIRTHS IN U.S.A. 1922–36.[1]

| | All | Twins | Triplets | Quadruplets |
|---|---|---|---|---|
| Total births .. | 28,244,869 | 630,460 | 9,123 | 196 |
| Live births .. | 27,338,663 | 588,810 | 8,010 | 158 |
| % still-births .. | 3·13 | 6·1 | 12 | 20 |
| % males, all births | 51·6 | 50·9 | 50·0 | 52·6 |
| % males, live births | 51·4 | 50·6 | 49·4 | 50·6 |
| Pregnancies .. | 27,920,030 | 315,230 | 3,041 | 49 |
| Frequency (all births) | | 1 : 88·6 | 1 : 9,150 | 1 : 570,000 |
| Cf. Veit 13,000,000 births[2] .. .. | | 1 : 80 | 1 : 7,910 | 1 : 371,126 |
| Berkeley, etc.[3] .. | | 1 : 90 | 1 : 7,628 | 1 : 670,734 |

"There are only fifty-one cases of quintuplets on record, thirty-two authenticated. All babies died: except in one group all died within the hour; in another only one lived (for fifty days); the third group is the Dionne family. There are well-authenticated cases of six and seven children born at one confinement, but all died."[3]

The third line of the table illustrates well one effect of multiple pregnancies: still-birth ratio is doubled with each additional fœtus. The next two lines show that, contrary to popular belief, the proportion of male babies is only very slightly less in multiple than in single pregnancies.

There is a very definite hereditary tendency to multiple pregnancies: either mother or father may be responsible. Mrs. G.'s mother's mother had identical twin girls, and Mr. G.'s mother's sister had boy-and-girl twins. One woman produced twins three times, triplets six times and twice quadruplets: she was one of quadruplets and her husband himself a twin. The Russian Wasilef had by his first wife quadruplets four times, triplets seven times and twins sixteen times: and by the second triplets twice, and twins six times. Another Russian peasant, Kirilow, had by his first wife quadruplets four times, triplets seven times, and twins six times. A Frenchman had triplets seven times by his wife and once by his mistress.[2]

*Influence of Sex.*[2] Uniovular or "identical" twins are little more than half as frequent (10/17) as binovular, resulting from fertilization of two ova.* Triplets can result from fertilization of one, two or three ova: the second is the most frequent, with one pair of identical twins and another baby from a second ovum. Uniovular quadruplets, etc., are rare: nearly all such multiple births result from fertilization of two or three ova, as in the case reported above. The Dionne quintuplets were *probably* uniovular.

All uniovular twins are like-sexed: but in addition more than half binovular twins are like-sexed, as though difference in sex were a factor inimical to survival. A similar tendency occurs in triplets, where unlike sex is unusual and still more in quadruplets, etc., where it is rare.

Survival brings out a definite difference between the sexes. The neonatal mortality is much greater for twins than for single births: for female twins it is about 12 per cent.; for males nearly 50 per cent., four times as great. The difference is greater as the number of babies increases: hence very few males survive from quadruplet, etc., births.

"It is a popular opinion . . . that if twins be of different sexes, the female is sterile". Simpson[5] investigated the possibility of the occurrence in humans of a sterile intersex female, analogous to the free-martin in cattle: and showed that there is no ground for belief that such a condition occurs in humans. Of 123 women born co-twin with males 112 had families, i.e. only 10 per cent. of their marriages were infertile—exactly the figure for marriages of single-birth women in the same area: the co-twin mothers averaged slightly more children than the others.

From Dr. A. R. Dafoe's description of the birth of the Dionne quintuplets:[4] "At 4 a.m., May 28th (1934) a 'hurry' call came from the Dionne home . . . two babies had already been born and a third was just making its appearance over the perineum. A kettle of water boiling was the sole preparation for the labour. I took over the situation and delivered the third baby. In the meantime another amniotic sac

* Crew, F. A. J., gives proportions which differ considerably from these. *Practitioner,* 1947, i, 233.

was presenting itself at the vaginal orifice, and a little pressure over the abdomen brought another baby into the world. This one was followed by still another. The last two babies were born within intact amniotic sacs. . . . Apart from shock and some post-partum hæmorrhage, and later a phlebitis in the right saphenous vein, the mother did well. The placenta was single, irregular in outline, with the cords emerging from it at various points : unfortunately it was destroyed. The babies appear to resemble one another considerably, and all are girls, still alive and thriving. Their combined weight at birth was 13 lb. 6 oz."

### REFERENCES

[1] Strandskov and Siemens, " Sex-Ratios at birth in U.S.A., 1922-36." *Amer. Jl. Physical Anthropology*, 1947, p. 491.

[2] Browne, F. J., *Antenatal and Post-natal Care*, London, 1946, p. 159.

[3] Berkeley, Bonney and Macleod, *Abnormal in Obstetrics*, London, 1937, p. 283.

[4] *Jl. A.M.A.*, 1934, Sept., p. 673.

[5] *B.M.J.*, 1949, 1, p. 49—quoting *Selected Obstetrical and Gynaecological Works of Sir J. Y. Simpson*, Edinburgh, 1871, p. 822.

## Figures and Tables

**Figure f1:**
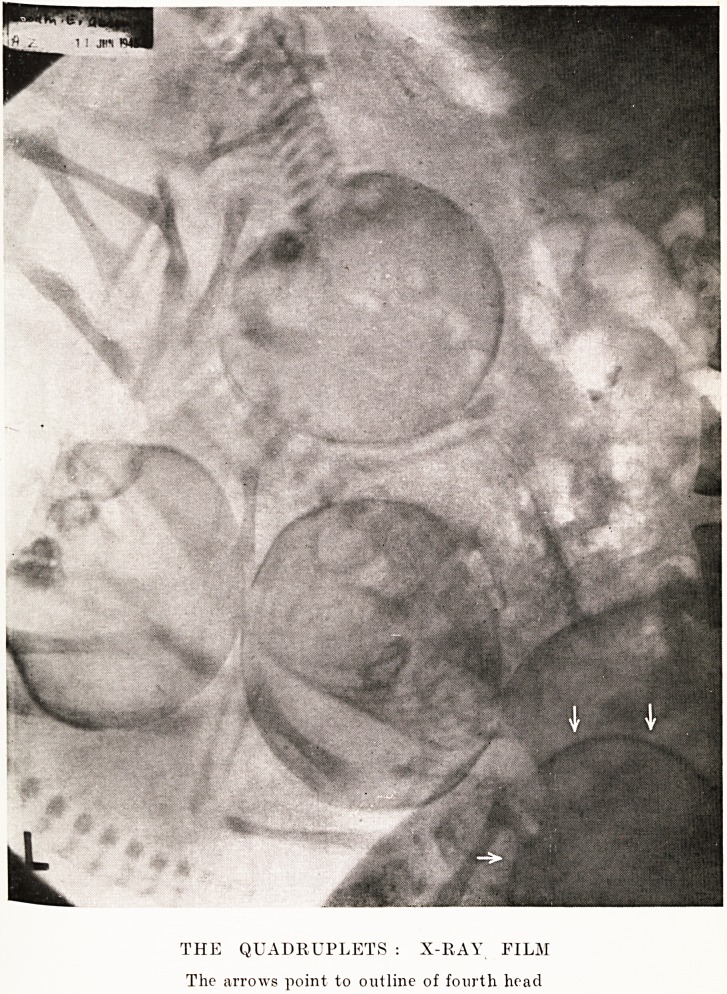


**Figure f2:**